# Whole-brain functional connectivity predicts regional tau PET in preclinical Alzheimer’s disease

**DOI:** 10.1093/braincomms/fcaf274

**Published:** 2025-07-15

**Authors:** Hamid Abuwarda, Anne Trainer, Corey Horien, Xilin Shen, Sophia Moret, Suyeon Ju, R Todd Constable, Carolyn Fredericks

**Affiliations:** Department of Neurology, Yale School of Medicine, New Haven, CT 06519, USA; Interdepartmental Neuroscience Program, Yale School of Medicine, New Haven, CT 06519, USA; Department of Neurology, Yale School of Medicine, New Haven, CT 06519, USA; Interdepartmental Neuroscience Program, Yale School of Medicine, New Haven, CT 06519, USA; Department of Biomedical Engineering, Yale School of Medicine, New Haven, CT 06519, USA; Department of Neurology, Yale School of Medicine, New Haven, CT 06519, USA; Department of Neurology, Yale School of Medicine, New Haven, CT 06519, USA; Interdepartmental Neuroscience Program, Yale School of Medicine, New Haven, CT 06519, USA; Department of Biomedical Engineering, Yale School of Medicine, New Haven, CT 06519, USA; Department of Radiology, Yale School of Medicine, New Haven, CT 06519, USA; Department of Neurology, Yale School of Medicine, New Haven, CT 06519, USA; Interdepartmental Neuroscience Program, Yale School of Medicine, New Haven, CT 06519, USA

**Keywords:** dementia, neurodegeneration, asymptomatic, multimodal, machine learning

## Abstract

Preclinical Alzheimer’s disease, characterized by the abnormal accumulation of amyloid-β prior to cognitive symptoms, presents a critical opportunity for early intervention. Past work has described functional connectivity (FC) changes in preclinical Alzheimer’s disease, yet the predictive *nature* between the functional connectome and Alzheimer’s disease pathology during this window remains unexplored. We applied connectome-based predictive modelling to investigate the ability of resting-state whole-brain FC to predict tau (18F-flortaucipir) and amyloid-β (18F-florbetapir) PET binding in preclinical Alzheimer’s disease (A4, *n* = 342 amyloid-β-positive, age 65–85). Separate models were developed to predict amyloid PET signal in the posterior cingulate, precuneus, and cortical composite regions, and to predict tau PET signal in each of 14 cortical regions that demonstrated meaningful tau elevation as identified through a Gaussian mixture model approach. Model performance was assessed using a Spearman’s correlation between predicted and observed PET binding standard uptake value ratios. We assessed the validity of significant models by applying them to an external dataset and visualized the underlying connectivity that was positively and negatively correlated to regional tau. We found that whole-brain FC predicts regional tau PET, outperforming FC-amyloid-β PET models. The best-performing tau models were for regions affected in Braak stage IV-V regions (posterior cingulate, precuneus, lateral occipital cortex, middle temporal, inferior temporal, and banks of the superior temporal sulcus), while models for regions of earlier tau pathology (entorhinal, parahippocampal, fusiform, and amygdala) performed poorly. Importantly, FC-based models predicted tau PET signal in the Alzheimer’s Disease Neuroimaging Intitative-3 dataset (amyloid-β-positive, *n* = 211, age 55–90) in tau-elevated but not tau-negative individuals. For the posterior cingulate tau model, the most accurate model in A4, the predictive edges positively correlated with posterior cingulate tau predominantly came from nodes within temporal, limbic, and cerebellar regions. The most predictive edges negatively associated with tau were from nodes of heteromodal association areas, particularly within the prefrontal and parietal cortices. These findings reveal that whole-brain FC meaningfully predicts tau PET in preclinical Alzheimer’s disease, particularly in regions affected in advanced disease, and are relevant across the Alzheimer’s disease clinical spectrum in individuals with elevated tau PET burden. This suggests that functional connectivity, likely in conjunction with other factors, may play a key role in early processes that facilitate later-stage tau spread. These models highlight the potential of the functional connectome for the early detection and monitoring of Alzheimer’s disease pathology, especially in later-stage target regions.

## Teaser

Abuwarda *et al*. demonstrate that whole-brain functional connectivity predicts regional tau tracer binding in preclinical Alzheimer’s disease. Their models showed highest accuracy for Braak stage IV–V regions but limited predictive power for early Braak I–III regions.

## Graphical Abstract

**Graphical Abstract:**
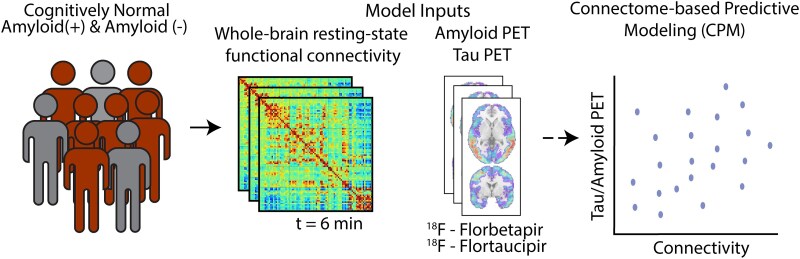

## Introduction

Alzheimer’s disease is a progressive neurodegenerative process characterized by the build-up of amyloid-β and tau pathology. Amyloid-β spreads quickly and diffusely throughout the neocortex, a process which can begin decades prior to symptom onset.^1,2^ In amnestic Alzheimer’s disease, tau spread parallels clinical symptomatology and occurs in a stereotyped manner, following Braak staging.^3^ Cortical tau build-up typically begins in the entorhinal cortex, then spreads to the medial and lateral temporal cortices and cingulate gyrus, corresponding with the first overt cognitive changes. The later stages of the disease are subsequently characterized by tau spread to the remaining neocortex, when Alzheimer’s disease dementia begins^3-5^.

Alzheimer’s disease is also associated with characteristic changes in functional connectivity (FC). The default mode network (DMN), which is associated with short-term memory function and self-referential processing, shows reduced connectivity in amnestic Alzheimer’s disease.^6-9^. This is preceded by periods of hyperconnectivity in the DMN and regional connectivity changes, namely in early Braak stage regions.^10,11^ These characteristic DMN changes may align with the cascading network failure hypothesis, which proposes that overtaxed, highly connected hubs initially compensate for pathology but subsequently undergo network collapse, leading to dementia.^12^ However, while correlation- and regression-based studies that focus on these regions and networks are insightful and will advance our knowledge of the disease, they may also overlook important features of the disease due to the limited scope of analysis.

Importantly, the relationship between preclinical Alzheimer’s disease pathology and associated changes in the functional connectome is not well known. Evidence from animal models and emerging evidence from human studies suggest amyloid-β induces a state of hyperexcitability^13-16^ and hyperconnectivity.^12,17^ Amyloid-β deposition has been noted to overlap significantly with the DMN especially in the very early stages of Alzheimer’s disease. On the other hand, tau appears to travel along paths of FC^18-20^. To further our understanding of this relationship, an unbiased, data-driven approach is needed. Predictive models that incorporate features from the entire functional connectome can help address these gaps, particularly because cross-validation^21,22^ procedures reduce in-sample inflation, which is critical for studies with potential clinical applications.^23^ Accordingly, we used predictive modelling to evaluate whether the functional connectome can predict amyloid-β and tau PET signal in preclinical Alzheimer’s disease. Although the relationship between FC and pathology is complex and likely bidirectional,^24^ predictive models identify statistical relationships agnostically, without requiring assumptions of directionality or causality. These identified patterns can inform hypotheses for future mechanistic studies and potentially help better detect early Alzheimer’s disease connectivity changes which may not be reflected by other modalities. We focus on the preclinical phase of Alzheimer’s disease, as it offers an opportunity to visualize tau and amyloid-β binding prior to widespread pathology and neuronal death; preclinical Alzheimer’s disease may also be a prime window for intervention.

We investigated whether whole-brain FC contains predictive information for focal and global tau and amyloid-β PET tracer binding. To address this question, we used connectome-based predictive modelling (CPM), a machine learning technique that identifies edges which correlate with outcomes of interest.^25^ One advantage of CPM is it identifies conserved functional features across individuals, which can complement individual-specific studies of a disease process. Another major advantage is it incorporates information from the entire functional connectome, rather than limiting the scope of analysis to a specific network or region. CPM has typically been implemented to use the functional connectome as a predictor of cognitive^26-29^ and behavioural/symptomatic measures^30-33^. Here, we use CPM instead to assess to what extent the functional connectome can predict focal or global amyloid-β or tau binding, yielding insights into the relationship between connectivity and early-stage Alzheimer’s disease pathology.

Because tau has been shown in a growing body of literature to propagate between functionally connected regions,^18,20,34^ while amyloid-β shows more widespread associations with connectivity changes,^35^ we hypothesized that FC will better predict regional tau deposition.

## Materials and methods

### Datasets

The Anti-Amyloid Treatment in Asymptomatic Alzheimer’s disease (A4) study is a clinical trial study of cognitively unimpaired adults (aged 65–85).^36^ All downloaded imaging data and associated metrics correspond to baseline measurements acquired before any treatment intervention. Specific study inclusion and exclusion criteria are described elsewhere.^36^ Of those with baseline resting-state functional magnetic resonance imaging (rsfMRI), 1490 participants met the requisite motion threshold (<0.3 mm mean frame-to-frame displacement, total excluded = 158). Of those with available baseline rsfMRI, 393 subjects had tau PET images (*n* = 51 healthy controls, *n* = 342 amyloid-β-positive individuals). Site information is unavailable for A4. We use the amyloid-β-positive individuals in the A4 dataset as a discovery dataset to generate our predictive models. Demographics are found in Table 1.

**Table 1:** A4 and ADNI amyloid-positive participant demographics

	A4 (*n* = 342)	ADNI (*n* = 211)
Age, years (*μ* ±SD)	72.2 ± 4.9	75.7 ± 7.2
Females, *n* (%)	200 (58.5%)	110 (52.1%)
Education, yrs (*μ* ±SD)	16.2±2.7	16.2± 2.5
MMSE (*μ* ± SD)	28.6 ±1.3	27.1 ± 3.1
Global Amyloid, Centiloids (*μ* ± SD)	64.0± 32.7	74.4± 41.8
MCI count, *n* (%)	–	75 (35.6%)
Dementia count, *n* (%)	–	47 (22.3%)

The Alzheimer’s Disease Neuroimaging Initiative-3 (ADNI-3) is an observational cohort study enrolling participants from across the full spectrum of Alzheimer’s.^37^ Of those with fMRI, we removed 161 participants with multi-band fMRI due to poor data quality. 496 participants met the requisite motion threshold (<0.3 mm mean frame-to-frame displacement). Of those who were amyloid-β-positive, eight had missing tau PET metrics, leaving 211 individuals (from 45 study sites). We use the ADNI dataset as an external test dataset for models generated in A4. Demographics are found in Table 1.

### Image parameters

#### A4

Participants underwent scanning using a 3T MRI and included one resting-state fMRI scan acquired through an echo-planar imaging, 2D gradient-recalled, or gradient-recalled echo-planar imaging sequence (*t* = 6.5 mins, TR = 2925–3520 ms, TE = 30 ms, flip angle = 80–90°), and a T1-weighted anatomical (magnetization-prepared rapid gradient echo [MPRAGE]) scan (TR = 2300–7636 ms, TI = 400–900 ms, flip angle 9–11°). Participants were imaged using GE medical systems, Philips medical systems, and Siemens scanners. Details can be found on the A4 LONI Image and Data Archive site.

#### ADNI

Imaging consisted of a rsfMRI echo-planar imaging-BOLD sequence (*t* = 10 min, TR/TE = 3000/30 ms, flip angle = 90°). MPRAGE anatomical sequences were also obtained (TR = 2300 ms, TI = 900 ms, flip angle = 9°). Participants were imaged using GE medical systems, Philips medical systems, and Siemens scanners. Details can be found on the ADNI LONI Image and Data Archive site.

### MRI preprocessing

A detailed description of data preprocessing is outlined in previous work.^38^ Briefly, MPRAGE scans were skull stripped using optiBET^39^ and nonlinearly aligned to the MNI-152 template using BioImage Suite^40^. Functional images were slice-time corrected (except those from Philips scanners as no slice-time parameters were provided) and motion corrected using SPM12. Scans with a mean frame-to-frame displacement >0.3 mm were excluded to minimize motion-related artefacts.^27,38^ Linear, quadratic, and cubic drifts; a 24-parameter motion model^41^; and signals from cerebrospinal fluid, white matter and global regions were statistically removed from the data as detailed previously.^42^ Global signal was regressed from all matrices. Region-of-interest (ROI)-to-ROI pairwise correlations were calculated using Pearson correlation coefficients, with all correlation values (both positive and negative) retained in resultant connectivity matrices. Negative correlations were preserved to maintain as much information as possible for predictive models, as the sign of connectivity relationships is less critical than their collective predictive power. MRIs across datasets were processed similarly. ROIs were removed from all connectivity matrices if at least >50 participants had signal dropout (Supplementary Table 1); this resulted in removal of 15 ROIs.

### PET preprocessing

For A4, tau PET ([18F]flortaucipir/FTP) standardized uptake value ratios (SUVR) measurements were obtained from A4, using the 90–110 min window post-injection (4 × 5-min frames). Preprocessing and analysis were performed using PETSurfer, an implementation offered in FreeSurfer (v. 6.0+). Each of the 5-min tau PET frames were motion-corrected and then averaged. These composite PET images was registered to corresponding MRI images, segmented according to the Desikan–Killiany atlas, and corrected for partial-volume effects using FreeSurfer. For each brain region defined by the Desikan–Killiany atlas, average tracer uptake values were calculated and standardized against the whole cerebellar cortex as the reference region, generating SUVRs. For amyloid-β PET, global amyloid-β centiloid and regional amyloid-β SUVRs (florbetapir-PET/FBP) were obtained from the A4 online data release (Version: 2021-04-01). Regional amyloid analyses were limited to precuneus and posterior cingulate (PCC) cortex regions, as these were the only regions with both available amyloid data in the A4 dataset and corresponding elevated tau signal suitable for models. Amyloid-β metrics were derived using 50–70 min (4 × 5-min frames) post-injection images. Images were summed and realigned into a single 3D image. SUVRs were computed using the whole cerebellum as the reference region.

For ADNI, partial-volume corrected tau PET ([18F]flortaucipir/FTP) metrics were derived from the ADNI study’s records using 75–105 min (6 × 5 min frames) post-injection images. Images were coregistered and parcellated as described above. SUVRs were computed using the inferior cerebellar cortex as the reference region.

#### Selection of ROIs

For tau analysis, we initially evaluated all 34 bilateral cortical ROIs and the amygdala in the Desikan–Killiany atlas in the A4 cohort. To identify regions with meaningful tau accumulation, we modelled all ROIs using a two-component Gaussian mixture model, allowing variance parameters to vary between components and assuming the abnormal Gaussian is the component with the higher mean. Regions where a two-component Gaussian distribution fit better than a one-component model (as evaluated by Bayesian Information Criterion) were assumed to have meaningful tau build-up. Of the 35 total ROIs evaluated, 26 showed superior fit with the two-component model. Upon visual inspection, we excluded three additional regions that deviated from the expected distributions of abnormal/elevated tau in preclinical Alzheimer’s disease. We calculated the mixing proportion of the abnormal component to estimate the number of individuals who would be considered tau-elevated in each region. ROIs with fewer than 20 estimated abnormal cases were removed to ensure sufficient signal for modelling tau patterns. Results of this can be found in Supplementary Table 2. In addition to the 14 remaining ROIs derived in this manner, we included a temporal meta-ROI (comprised of entorhinal, parahippocampal, amygdala, fusiform, middle temporal and inferior temporal regions) previously described in the literature.^43^ For amyloid-β, we modelled an amyloid-β cortical composite SUVR. We also included the PCC and the precuneus (PreC) amyloid-β SUVR to enable direct one-to-one comparisons with tau SUVR metrics in these same regions.

#### Tau z-scores

For regions demonstrating significant predictions in bilateral ROIs, we calculated tau z-scores using the healthy control cohort (*n* = 51) as a normative reference. We fit a linear regression model with age and sex predictors to tau SUVR values in this control group, and then applied this model to amyloid-β-positive participants to quantify their deviation from the expected tau SUVR for matched age and sex. *Z*-scores were derived from these deviations to standardize the measurement of pathological tau burden relative to healthy controls. Additionally, we conducted separate analyses for hemisphere specific tau in these regions, developing distinct models for right and left-hemispheric SUVRs.

### Connectome-based predictive modelling

To identify the edges predictive of focal or global tau or amyloid-β in each model derived as above, we employed CPM, the specifics of which can be found in Shen *et al.*^25^ Briefly, FC matrices (predictors) and PET metrics (outcomes) were compiled for each participant. For each cell in the connectivity matrix (representing correlation of activity between each pair of nodes), the edge strength of all participants is aggregated and then correlated with PET tracer metrics (amyloid-β and tau, both focal and global). This process was repeated for every connection in the brain, resulting in a single correlation matrix for the study, where each cell represents the correlation of the connectivity to the PET metrics in the dataset. We use a partial correlation approach in which scanner and a mean frame-to-frame displacement are covariates when generating the study-wide correlation matrix. The connections that demonstrated a significant correlation to the outcome were selected for. For each participant, the significant connections from their connectivity matrix were summed to generate a patient-specific brain network summary score. All participants’ summary network scores were related to the outcome measure through a linear regression to model the ‘brain-outcome measure’ relationship. The linear regression model was validated using a 5-fold cross-validation. This process was repeated 1000 times, where each time, individuals were randomly re-shuffled into new groups, generating an unbiased estimation of the study cohort.

Models were rerun with different edge-selection thresholds (*P* = 0.01, *P* = 0.05), with minimal difference in predictive power (Supplementary Table 3).

### Statistical analyses of models

Model performances were assessed by using a Spearman’s correlation between predicted and true PET metrics. In addition, we tested our models against permuted models, whereby the participant labels were randomly shuffled before running the model. After iterating this random permutation 1000 times, we calculated how often the accuracy of the randomly permuted predictions surpassed the median accuracy of the regular predictions. This was used to calculate a non-parametric *P*-value, described in Scheinost *et al*.^44^


(1) $$P\;=\;\frac{\#\{\rho_{\mathrm{null}}\geq \rho_{\mathrm{median}}\}}{1000}$$

Significant models were then tested against a spatially permuted model, where each individual was assigned one of their own tau values randomly selected from the 14 individual ROIs as model input. Significance was determined as defined by Equation (1). We adjusted these non-parametric *P*-values for multiple comparisons using the Benjamini–Hochberg procedure.

### Post-hoc analysis

#### Jaccard index

To quantify similarity between predictive features across models, we generated binary masks of the study-wide correlation matrix in CPM, indicating the presence (1) or absence (0) of edges significantly correlated with each PET metric. To ensure robustness, we only retained edges that were selected in at least three out of 5-folds and appeared in a minimum of 600 of the 1000 iterations. The Jaccard similarity index was calculated between feature sets as the ratio of the intersection (significant edges present in both models) to the union (significant edges present in either model). This metric ranges from 0 (no overlap) to 1 (complete overlap), providing a direct measure of feature similarity across model feature sets.

#### Lesioning analyses

Temporal lobe regions were defined using the Broadman atlas, while DMN nodes were identified using a group-wise normalized cut algorithm,^45^ yielding 10 networks including a DMN consistent with established definitions. Lesioning was conducted by removing: (i) all temporal nodes, (ii) all DMN nodes, and (iii) both temporal and DMN nodes. For each of these analyses, connectivity edges from these nodes were excluded from the models, and CPM was rerun.

#### Model visualizations

Following previous studies, edges were visualized if they were found to be significant in a minimum of three out of five folds in 600 of the 1000 CPM replicates, which helps reduce noise while preserving important connections.^26,31,38^ To visualize the most predictive nodes and their associated predictive edges, we generated a mask where significant correlations were represented by 1’s and non-significant edges by 0’s. From this binary mask, we calculated each node’s degree by summing the number of significant edges. We then selected the top 5% of nodes based on this degree and visualized their significant connections using circle plots and brain surfaces. Circle plots were used to depict these edges, where each node is represented by a circle at the edge of the plot, representing all 268 nodes from the shen-268 atlas, with lines indicated an edge between two nodes. Brain plots show the same edges, plotted onto the brain.

### Model generalization

Thresholds for determination of tau negativity versus tau-elevation were established as the SUVR value at which the posterior probability of belonging to the normal (lower mean) component exceeded 99%, as previously done.^46^ We used edges that were significant in a minimum of three out of five folds in 600 of the 1000 CPM replicates within the A4 dataset. These edges, along with the corresponding beta-values from linear regression models derived from A4, were directly applied to the ADNI connectivity matrices. This process was separately applied to tau-negative and tau-elevated individuals. Generalization was assessed by Spearman’s correlation of predicted and true tau PET metrics of ADNI participants.

## Results

### FC minimally predicts local and global amyloid PET binding

We set out to determine whether FC could predict amyloid-β PET. We developed a model to predict global amyloid-β, given its use in clinical and research settings in determining amyloid-β positivity. We found CPM minimally predicted global amyloid-β (Supplementary Fig. 1; $r_{s}$ = 0.04, *P* = 0.41, FDR-adjusted for three tests). To assess whether CPM might better predict regional amyloid SUVR over a more global measure of amyloid-β, we developed models to predict amyloid-β binding in the PCC and PreC, regions that demonstrate the earliest build-up of amyloid-β in preclinical Alzheimer’s disease.^47^ We did not find meaningful predictive power for amyloid-β PET SUVR values in these regions (Supplementary Fig. 1; PCC $r_{s}$ = 0.04, *P* = 0.41; PreC $r_{s}$ = − 0.03, *P* = 0.60; FDR-adjusted for three tests).

**Figure 1:**
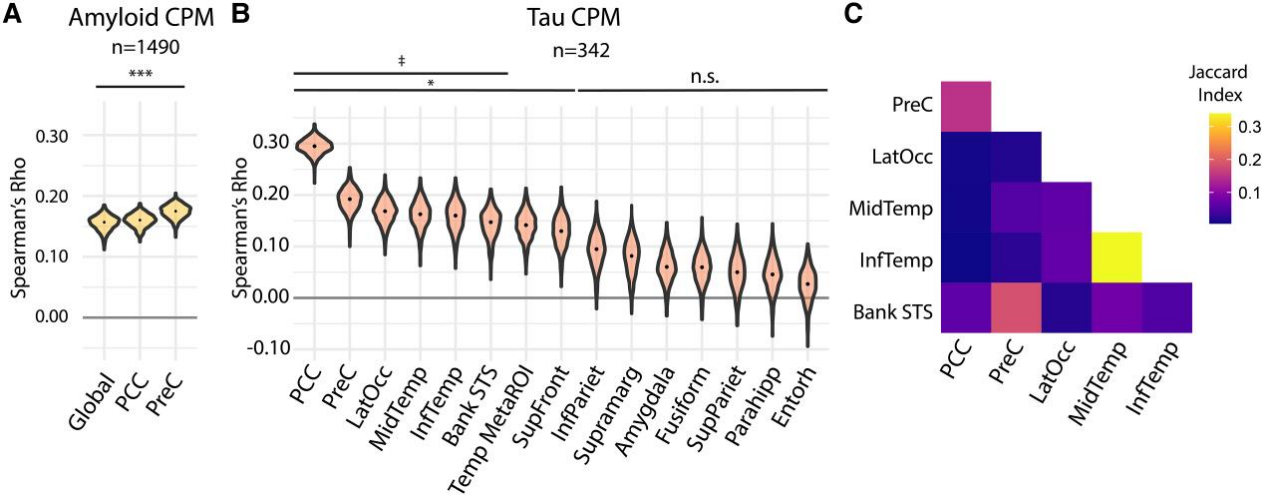
**Whole-brain FC predicts regional tau PET signal in preclinical Alzheimer’s disease. (A)** Amyloid CPM results, representing accuracies of whole-brain FC predicting amyloid PET SUVRs. Violin plots represent distributions over 1000 model iterations of global amyloid SUVR and regional amyloid SUVR in the PCC and PreC regions. Model performance is measured by Spearman’s correlation ($r_{s}$) of predicted versus observed amyloid PET. *n* = 1490 amyloid-positive and amyloid-negative participants. *** *P* < 0.001, false-discovery rate (FDR)-adjusted for 3 tests (1-tailed, permutation test). **(B)** Regional tau CPM results, representing accuracies of whole-brain FC predicting tau PET SUVRs. Violin plots represent distribution over 1000 model iterations, with the median model performance depicted by the black dot. Model performance is measured by Spearman’s $r_{s}$ of predicted versus observed regional tau PET. *n* = 342 amyloid-positive participants. * *P* < 0.05 against permutation testing; FDR-adjusted for 14 tests (1-tailed, permutation test). ‡ *P* < 0.05 against spatially permuted model, FDR-adjusted for seven tests (1-tailed permutation test). **(C)** Similarity of predictive edges between regional tau models, using the Jaccard index (intersection of edges divided by the union of edges present between each pair of models).

While this primary analysis focused on the cohort with tau PET imaging, we also generated amyloid models in the entire study population (*n* = 1490) to assess these findings across a full range of amyloid-β levels, including those below the threshold for amyloid-β positivity. We found these models modestly predicted amyloid-β PET signal (Fig. 1A; global amyloid-β $r_{s}$ = 0.16, *P* < 0.001, PCC $r_{s}$ = 0.16, *P* < 0.001, PreC amyloid $r_{s}$ = 0.17, *P* < 0.001).

### FC better predicts regional tau binding in preclinical Alzheimer’s disease

We next applied CPM, using whole-brain FC matrices as input, to predict regional tau binding. CPM predicted tau SUVR in the PCC, PreC, lateral occipital, middle temporal, inferior temporal, bank superior temporal sulcus (STS), and superior frontal ROIs (Fig. 1B, median CPM: PCC $r_{s}$ = 0.30, *P* < 0.001; PreC $r_{s}$ = 0.192, *P* < 0.001; lateral occipital $r_{s}$ = 0.17, *P* = 0.03; middle temporal $r_{s}$ = 0.16, *P* = 0.03; inferior temporal $r_{s}$ = 0.16, *P* = 0.03, bank STS $r_{s}$ = 0.15, *P* = 0.03; superior frontal $r_{s}$ = 0.13, *P* = 0.03; FDR-adjusted for 15 tests), with all other ROIs not significant against permuted models. To determine whether the models that survived permutation testing contained ROI-specific information or merely reflected global tau covariance, we generated a spatially permuted model in which each individual was assigned one of their own tau values randomly selected from the 14 individual ROIs as model input. All models except the superior frontal significantly outperformed this spatially permuted model (Fig. 1B, PCC *P* < 0.001; PreC *P* < 0.03; lateral occipital *P* = 0.04; middle temporal *P* = 0.04; inferior temporal *P* = 0.04, bank STS *P* = 0.04, superior frontal *P* = 0.06, FDR-adjusted for seven tests). When comparing the correspondence of the predictive edges between these models, we found that these tau models were spatially distinctive (Fig. 1C); decreasing the feature selection threshold to *P* = 0.01 resulted in more overlapping edges between models (Supplementary Fig. 2). There was no correlation between the prediction accuracy for a region and the estimated number of individuals in the tau-positive distribution for that region (*r* = −0.06). CPM tau models predicting tau z-scores (calculated relative to the 51 healthy control participants in A4/LEARN) were developed to assess potential contributions of non-specific/off-target signal to prediction accuracies, which accounted for normal age- and sex-related changes in healthy controls. These z-score models showed predictive accuracies comparable to models of raw SUVR. Additionally, modelling left and right hemispheric ROIs separately provided no additional predictive accuracy (Supplementary Table 4). FC versus tau scatterplots and model calibration plots can be found in Supplementary Fig. 3.

We next assessed to what degree our tau model predictions depended on: (i) connections within the temporal lobe, including regions which have elevated tau in the earliest stages of Alzheimer’s disease (Braak I–IV) and (ii) connections from the connectivity network most implicated in amnestic Alzheimer’s disease, the DMN. We lesioned all temporal nodes, all DMN nodes, and all temporal and DMN nodes in separate models (Supplementary Fig. 4), finding that all models maintained their predictive accuracies despite the lesions (Supplementary Fig. 5). This suggests that regional tau binding can be predicted using information from connectivity outside of temporal or DMN nodes.

In summary, regional tau models for the PCC cortex, PreC, lateral occipital, middle temporal, inferior temporal, and bank STS ROIs outperformed the global tau SUVR model. Additionally, when comparing equivalent regions, PCC and PreC tau ROI models outperformed their corresponding amyloid-β SUVR models, despite the higher statistical power available for the amyloid-β models.

### Tau models generalize to tau-positive patients in an external clinical dataset

**Figure 2:**
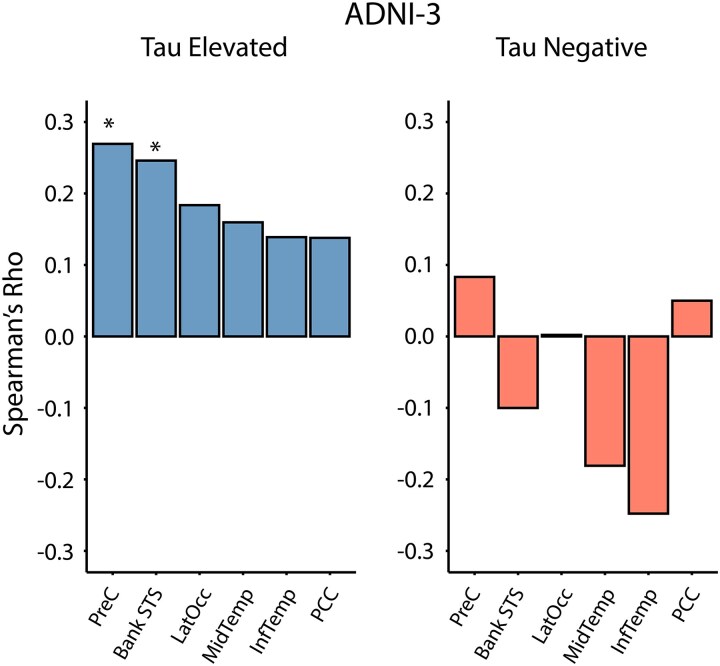
**Regional tau binding models generalize to an external dataset.** Validation of A4 regional tau models in the ADNI-3 dataset, differentiated by tau-elevated and tau-negative groups. Model performance is measured by Spearman’s $r_{s}$ of predicted versus observed regional tau PET in ADNI *(n* = 211). *P < 0.05 against permuted model, FDR-adjusted for six tests (1-tailed permutation test).

To assess the generalizability of our models, we sought to externally validate the six most predictive FC-based regional tau models in an external Alzheimer’s disease dataset, ADNI-3. ADNI-3 is a study that includes healthy controls, and participants with subjective memory concerns, mild cognitive impairment, and Alzheimer’s disease dementia. We restricted our generalization analysis to amyloid-β-positive individuals in ADNI, to ensure consistency with the amyloid-β-positive training population (A4) (Supplementary Fig. 6). To concomitantly test model specificity, we applied the CPM models separately to tau-negative and tau-elevated individuals, defined by a two-component Gaussian mixture model approach. A4 models modestly predicted tau levels in tau-positive individuals in ADNI-3 (Fig. 2; PCC $r_{s}$ = 0.14, *P* = 0.14; PreC $r_{s}=0.27,\;p=0.02;$ lateral occipital $r_{s}$ = 0.18, *P* = 0.052; middle temporal $r_{s}$ = 0.16, *P* = 0.052; inferior temporal $r_{s}$ = 0.14, *P* = 0.052; bank STS $r_{s}$ = 0.25, *P* = 0.006; FDR-adjusted for six tests), but not in tau-negative individuals (Fig. 2; PCC $r_{s}$ = 0.05, *P* = 0.68; PreC $r_{s}=0.08$, *P* = 0.68; lateral occipital $r_{s}$ = 0.00, *P* = 0.97; middle temporal $r_{s}$ = −0.18, *P* = 0.30; inferior temporal $r_{s}$ = −0.25, *P* = 0.09; bank STS $r_{s}$ = −0.10, *P* = 0.30; FDR-adjusted for six tests).

### PCC positive and negative predictive nodes and edges are spatially distinct

**Figure 3:**
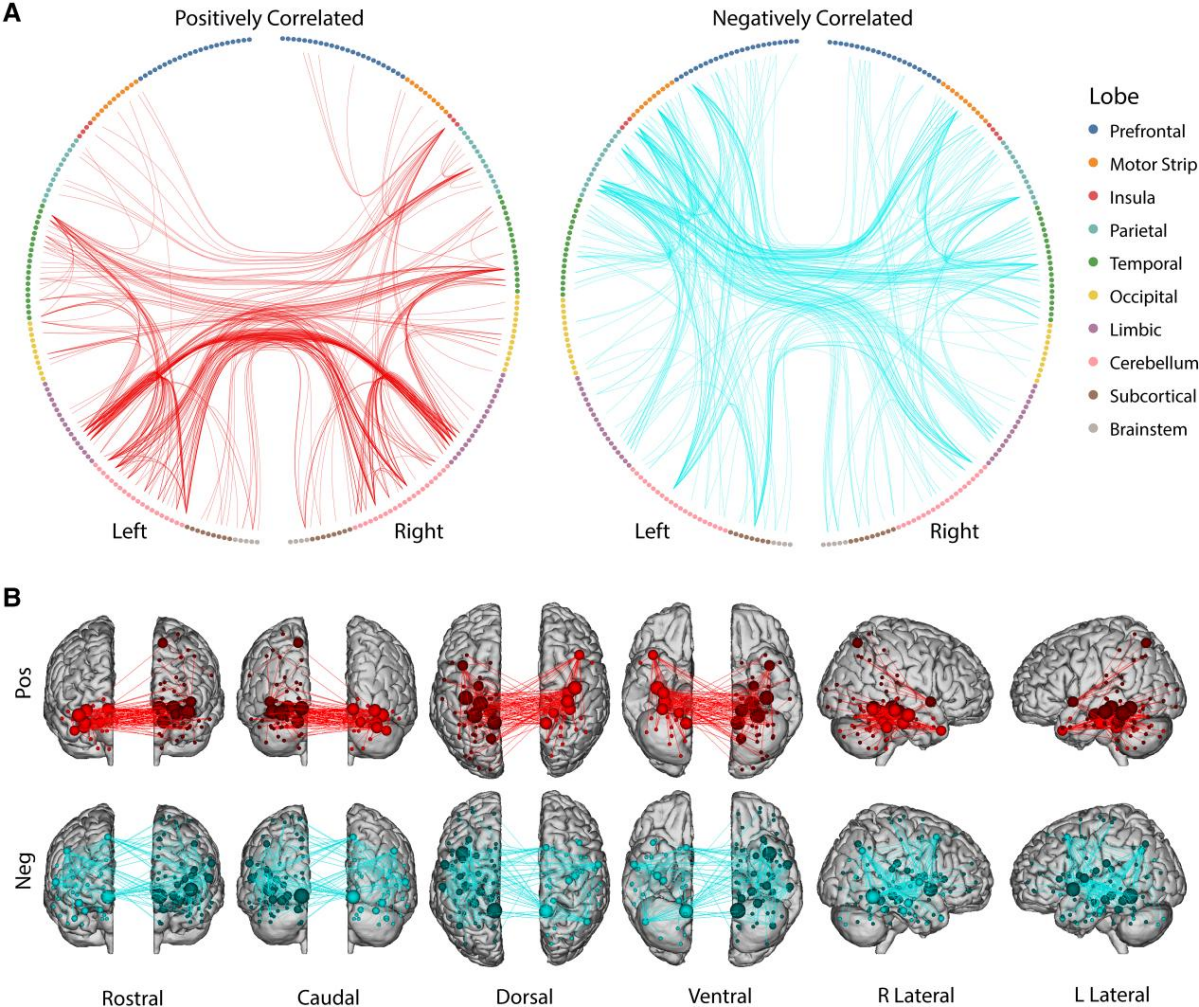
**PCC tau model edges positively and negatively correlated with FC.** (**A**) Circle plots of the predictive edges for the PCC model (*n* = 342). Each circle represents a node from the Shen-268 atlas, grouped according to their respective region. Edges are depicted by lines between each node pair. Plot with red lines (left) shows edges that were positively correlated with PCC tau, while the plot with blue lines (right) indicates edges that were negatively correlated with PCC tau. Only edges from the top 5% most predictive nodes (by degree) are displayed, based on edges significant in ≥ 3/5-folds across ≥ 600/1000 connectome-based predictive modeling iterations. (**B**) The same predictive edges from circle plots **A** plotted onto brain surfaces.

Given that the PCC model demonstrated the highest predictive power, we examined its underlying predictive edges and nodes in more detail. We induce sparsity to appropriately visualize edges by looking at the most predictive nodes, defined as the top 5% of nodes (by degree) among positively and negatively correlated edges. Positively correlated edges were enriched in nodes of the lateral and medial temporal lobe and cerebellum, with a particular enrichment in cross-hemispheric medial temporal connections (Fig. 3A and B). Negatively correlated edges on the other hand were enriched in higher cortical association areas of the prefrontal and parietal regions (Fig. 3A and B). Circle plots for remaining A4 significant models are shown in Supplementary Fig. 7. Nodes were also grouped by network definitions, with resulting edge matrices shown in Supplementary Fig. 8.

## Discussion

We use a whole-brain, data-driven approach to determine how the functional connectome predicts amyloid-β and tau PET binding in a preclinical Alzheimer’s disease cohort (the A4 study). We demonstrate that whole-brain FC-based models predict regional tau PET binding, but more poorly predict amyloid-β binding. Tau models were most accurate for Braak IV–V regions (PCC, PreC, lateral occipital cortex, middle temporal, inferior temporal, and bank STS). These models generalized to an external dataset (ADNI-3) including both symptomatic and preclinical Alzheimer’s disease individuals with Alzheimer’s disease. Notably, models predicted tau PET signal specifically in tau-positive and not in tau-negative members of the ADNI cohort, emphasizing these models are not meaningful in individuals without abnormal tau. The underlying predictive edges of the PCC model (the most accurate in A4) suggest that there is a spatial distinction between the most predictive positively correlated and negatively correlated nodes and their respective edges.

Our use of CPM represents a novel direction from its typical use. While CPM has traditionally been used to relate connectome patterns to behavioural and psychiatric measures, we uniquely apply it to predict well-defined, biologically validated metrics of early-stage Alzheimer’s disease pathology. Multimodal predictive modelling, especially where one modality is well-described and well-validated, allows for investigations of FC to be anchored in biologically informed ways. That is, because tau and amyloid-β PET are reliable and valid measures of underlying Alzheimer’s disease pathology, we can use PET as an indirect way to more accurately, and agnostically, assess connectivity differences that track with Alzheimer’s disease progression. Furthermore, by incorporating additional dimensions to our analyses, multimodal approaches may enhance sensitivity to uncover disease characteristics not discernible from unimodal analyses, which may help better identify clinically meaningful subgroups. With interpretable models such as CPM, wherein one can identify the edges and nodes predictive of the outcome, we can also generate new hypotheses to test in future mechanistic studies.

A key finding of this work was that models demonstrating significant predictions of regional tau PET were more accurate than those predicting temporal meta-ROI tau, regional amyloid-β and global amyloid-β. Recent work has demonstrated an association between whole-brain tau PET covariance and FC.^19,34^ We add to this existing literature by demonstrating FC can predict regional tau PET signal, but minimally predicts amyloid-β PET. This finding converges with demonstrations that tau progresses along pathways of FC,^18-20^ and is more closely associated than amyloid-β with disease outcomes such as cortical atrophy and symptomatology,^48-51^ where tau’s spatial distribution corresponds more directly to clinical phenotypes and patterns of neurodegeneration. We demonstrate that regional tau models convey meaningful region-specific information that is not captured by global tau covariance, outperforming spatially permuted models. Models predicting regional tau PET SUVR values also outperformed those using the temporal meta-ROI, suggesting that region-specific variation in the relationship between FC and tau burden is obscured when signals are averaged into a composite ROI. Future work should examine how these models improve using functional parcellations of tau PET to allow more direct comparisons of tau PET to fMRI.

Interestingly our models were more accurate in predicting tau for regions associated with Braak stages (IV–VI) than those corresponding to earlier Braak stages (I–III). One interpretation is that FC in later-stage regions may be particularly sensitive to tau deposition, such that even modest tau build-up may lead to disproportionate FC disruptions. Alternatively, tau deposition might be driven by FC shifts in these regions, which are known to participate in major networks (e.g. PCC and PreC of the DMN). Notably, when generalizing to the ADNI-3 dataset, we observed shifts in regional predictive performance (e.g. diminished PCC prediction but enhanced bank STS prediction), likely reflecting the dynamic nature of Alzheimer’s disease progression and different disease stages captured across cohorts. This variability is further supported by recent work applying CPM to familial Alzheimer’s disease, which corroborates our finding that PreC tau can be predicted from FC.^52^ However, unlike our preclinical Alzheimer’s disease models, this study also found high predictability of entorhinal tau—a difference that may reflect the distinct disease trajectories between sporadic and genetic Alzheimer’s disease patients.

Our lesioning analyses highlight the distributed and heterogenous nature of predictive information across the brain—when key regions are lesioned, the model is likely using other relevant connectivity patterns that may shift in compensatory ways across remaining regions. This distributed redundancy may explain why lesioning nodes of the temporal lobe and DMN does not substantially impact model performance. When examining the underlying predictive edges for the PCC model, we saw a mixture of positively and negatively correlated edges associated with regional tau SUVR; the positively correlated edges from the most involved nodes were enriched in the temporal lobe, limbic, and cerebellar regions, whereas negatively correlated edges were enriched in nodes of heteromodal association cortices such as the prefrontal and parietal regions. Edges positively correlated with regional PCC tau may reflect compensatory connectivity or pathological hyperconnectivity patterns. The latter would be consistent with recent evidence suggesting that amyloid deposition in posterior DMN regions triggers hyperexcitability, which in turn drives aberrant connectivity between these regions and the temporal lobe.^14,53^ This amyloid-induced hyperconnectivity may facilitate tau propagation from its initial sites in the MTL to downstream cortical targets to directly impact cognition, particularly in parietal regions like PCC and PreC.^12-14,34,54^ Our finding that the most predictive models are for these regions aligns with this proposed mechanism and is consistent with the hypothesis that PCC and PreC are key early nodes for connectivity-mediated tau spread. Edges negatively correlated with regional PCC tau may represent a loss of normal connectivity. However, more work will be needed to more precisely distill these associations. Future work should also establish whether these identified edges may inform our ability to identify at-risk, clinically meaningful subgroups in preclinical Alzheimer’s disease.

Although this work generated generalizable models of preclinical Alzheimer’s disease from a single six-minute resting-state fMRI scan, future datasets that include longer scan durations, task-based fMRI paradigms (e.g. continuous performance tasks), correction for susceptibility-induced distortions, and leverage second-generation PET tracers and advanced PET imaging technologies could further enhance FC estimates and predictive performance.^27,55^ However, while these may help improve accuracies, the modest prediction metrics of tau suggest that FC alone may not sufficiently explain all of the variance in tau pathology. There are other caveats to the current work. While our study focused on amyloid-positive individuals and identified regions with meaningful tau accumulation using Gaussian mixture model, our models do not account for different baseline spatial patterns of tau across participants, which could potentially influence the relationship between FC and tau. The A4 study restricted enrollment to individuals who performed within 1.5 s.d. of the normative mean on a test of short-term memory.^36^ This may impact model generalizability in participants whose cognitive measures deviate from the average. Encouragingly, however, our results generalize to an external dataset with individuals with a broader range of clinical disease and cognitive test performance, suggesting they may be relevant regardless of cognitive performance. This highlights the utility of neuroimaging to detect and track preclinical Alzheimer’s disease pathophysiology, even in the absence of overt cognitive changes. FC may be an important complement to cognitive screening and plasma/CSF biomarkers in predicting trajectories in preclinical Alzheimer’s disease.

While CPM can help generate new hypotheses to better understand the disease, the ability to define causal relationships with predictive models is limited. Importantly, this predictive modelling approach does not imply the unidirectional relationship between FC and tau pathology. Our findings should therefore be further explored through quasi-experimental statistical techniques and animal experiments.^56^ For example, one such quasi-experimental technique, regression discontinuity, could be used to explore connectivity changes above and below certain critical tau thresholds in individuals given amyloid-β/tau clearing agents, helping to determine the causal effects of Alzheimer’s disease pathology on connectivity. A difference-in-differences approach could compare the change in FC over time in immediately adjacent regions with high and low tau accumulation, estimating the causal impact of tau on connectivity changes. Animal experiments can also causally assess some of the hypotheses we pose here. For instance, to determine whether the positively correlated edges we identify here are compensatory (Fig. 3A and B), researchers could study dose-dependent concentrations of tau in murine temporal ROIs while simultaneously using optogenetic inhibition of these ROIs during a memory task; if they are compensatory, the group with inhibition would experience earlier task-associated deficits compared to the control group.

Additionally, the calibration plots highlight that predicted tau values cluster within a narrower range than observed values, deviating from the identity line despite showing significant correlations. This clustering may biologically represent atypical participants, as we do not anticipate high tau SUVRs at this preclinical stage. This also highlights a methodological limitation of population-level models: their tendency to regress predictions towards the population mean, underestimating individuals with the highest tau burden. However, model failure itself may simultaneously present an opportunity to identify meaningful subpopulations.^57^ Participants whose tau accumulation is poorly predicted by connectivity-based models might share other complex disease features that could inform more personalized approaches. While exploring these patterns of model failure is beyond the scope of the present study, it represents a promising direction for future research to identify distinct trajectories in preclinical Alzheimer’s disease.

It will also be vital to more precisely assess the temporal aspects of disease progression. Past work has shown models defined across an entire sample might not be relevant for all individuals in the dataset.^57^ This is especially important in Alzheimer’s disease, which exhibits significant spatiotemporal heterogeneity. While amyloid-β positive individuals are at high risk for future Alzheimer’s disease development, asymptomatic amyloid-β positivity is a large window. Initial biomarker evidence of amyloid accumulation, such as elevated amyloid PET SUVR or decreased CSF Aβ42/Aβ40 ratios, can emerge approximately 10–20 years before the estimated onset of clinical symptoms.^2^ Better defining clinically meaningful subgroups within this preclinical Alzheimer’s disease stage may aid in future risk assessment and treatment selection. Techniques such as the subtype and stage inference algorithm (SuStain), which pseudotemporally assigns individuals into different stages of the disease while simultaneously clustering individuals based on similarities in disease progression,^58,59^ may address the limitation of cross-sectional Alzheimer’s disease analyses. Future longitudinal studies can also help distinguish temporal heterogeneity of individual patients across all forms of Alzheimer’s disease. The emergence of large preclinical datasets such as A4 represent a significant milestone in the field, allowing us to use robust statistical techniques to model preclinical illness for the first time. The potential to identify individuals with preclinical disease using highly sensitive and specific blood-based biomarkers will aid immensely in the early identification and, ultimately, treatment of Alzheimer’s disease.^60^

Our results underscore the ability of the functional connectome to predict regional tau deposition even in the preclinical stages of Alzheimer’s disease. It is striking that we can best predict tau PET binding in highly interconnected nodes that are associated with the DMN and with Braak stages IV and V pathology, such as the PCC, PreC, and lateral occipital regions. We hypothesize that this suggests major brain hubs (some of the strongest of which are part of the DMN) may play a critical role in the dissemination of tau in preclinical Alzheimer’s disease. Combining advanced neuroimaging techniques with computational approaches has the potential to identify Alzheimer’s disease progression, classify individuals into Alzheimer’s disease subgroups, and potentially reveal new therapeutic targets for personalized intervention in the earliest stages of illness.

## Supplementary Material

fcaf274_Supplementary_Data

## Data Availability

Data from the A4 and ADNI-3 studies can be requested online. Code for CPM scripts are available at https://github.com/orgs/frederickslab/repositories.
