# Supplementary material for: Whole-brain functional connectivity predicts regional tau PET in preclinical Alzheimer’s disease
Source: Brain Commun. 2025 Jul 15;7(4):fcaf274. doi: 10.1093/braincomms/fcaf274 (PMC12305425; doi:10.1093/braincomms/fcaf274)
Supplement: fcaf274_Supplementary_Data [file fcaf274_supplementary_data.docx]

# Supplemental Material

**
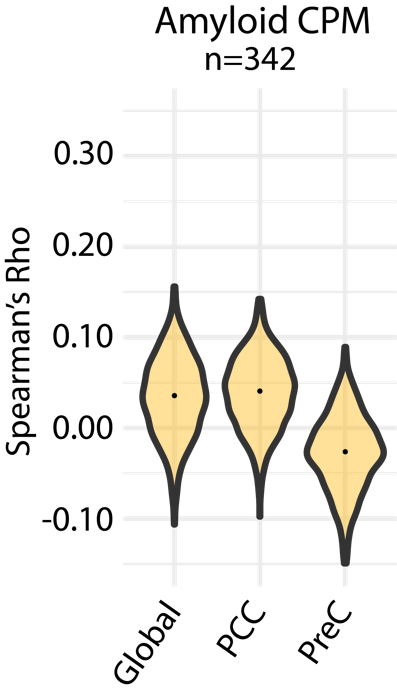
**

Supplemental Figure 1. Connectome based predictive models (CPM) models of amyloid SUVR for tau PET cohort (n = 342, amyloid-positive). Violin plots represent distribution over 1000 model iterations, with the median model performance depicted by the black dot. Global = global composite; PCC = posterior cingulate cortex; PreC = precuneus

**
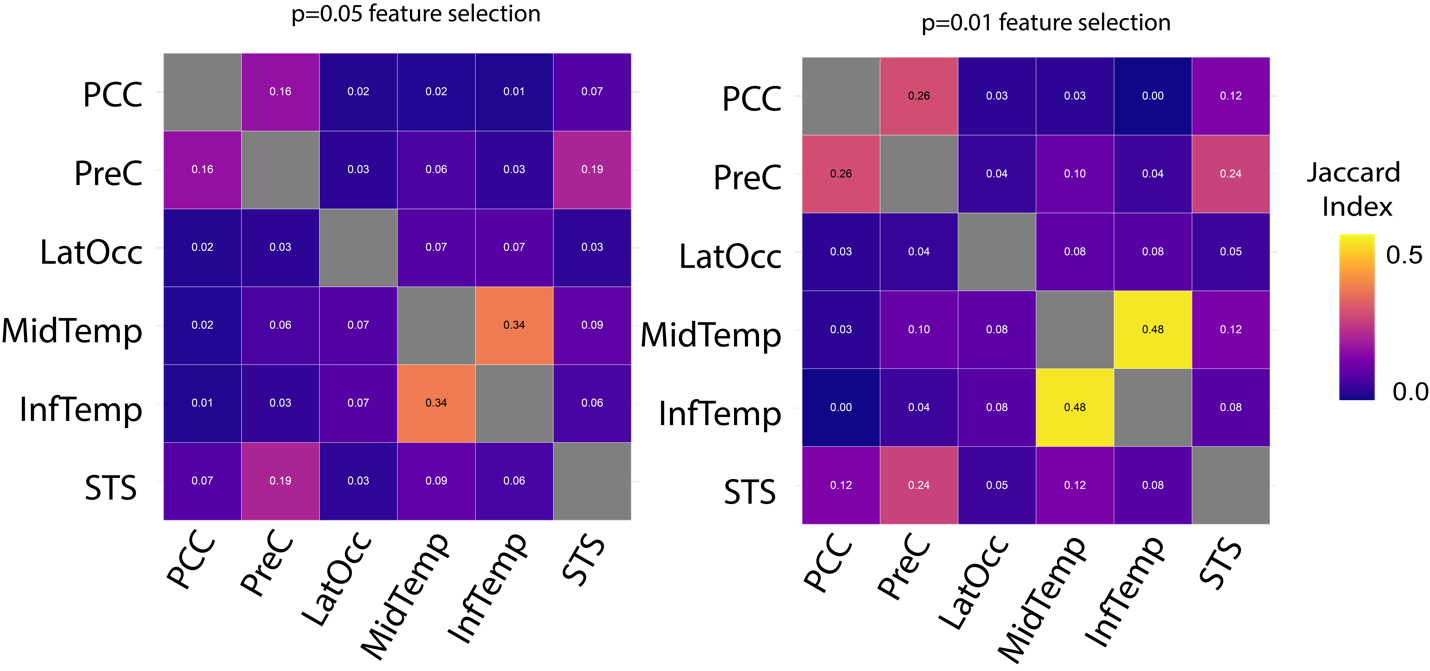

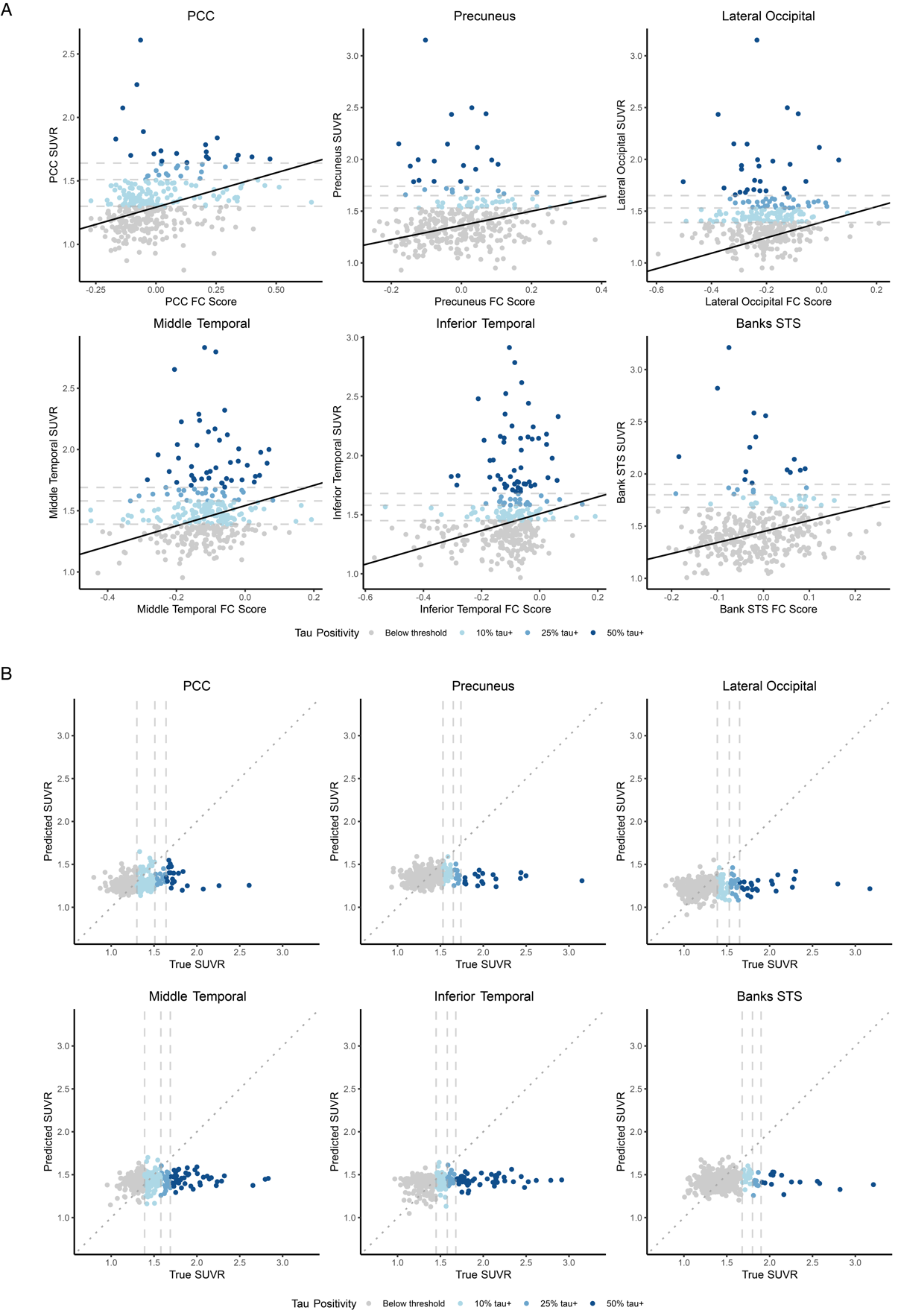
**

Supplemental Figure 2. Jaccard indices, representing the overlap of predictive features between pairwise models. Models were generated using either a p=0.05 feature selection threshold (left) or p = 0.01 threshold (right). Higher index values represent the models had more similar features.

Supplemental Figure 3. A4 model scatterplots and calibration plots. A) Scatterplots of functional connectivity (FC) score vs region-of-interest (ROI) SUVR for significant models. Lines defined by regression parameters from connectome-based predictive models (CPM, n=342). Each point represents a participant. B) Calibration plots of true tau standard uptake value ratio (SUVR) vs. predicted tau SUVR. For both panels, participants are colored by tau positivity probability, determined by the gaussian mixture model posterior probability of belonging to abnormal component. Each point represents a participant.

**
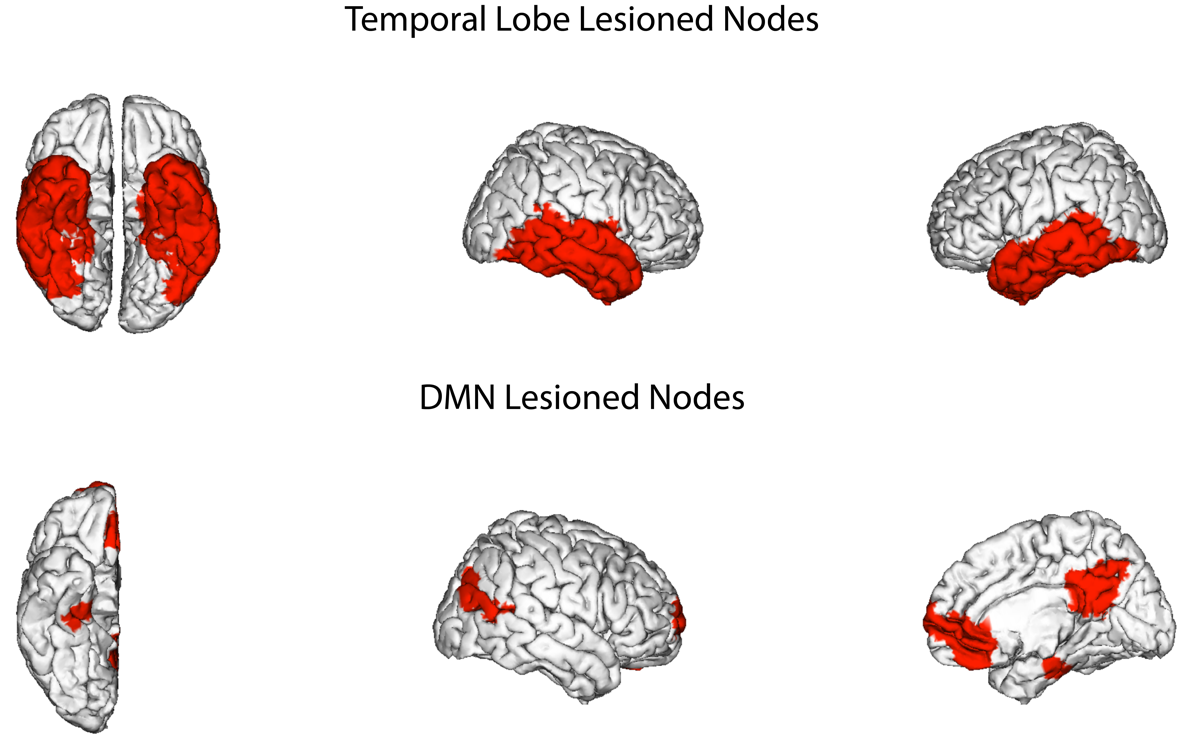
**

Supplemental Figure 4. Visualization of nodes of the temporal lobe and default mode network (DMN) used for lesioning analyses.

**
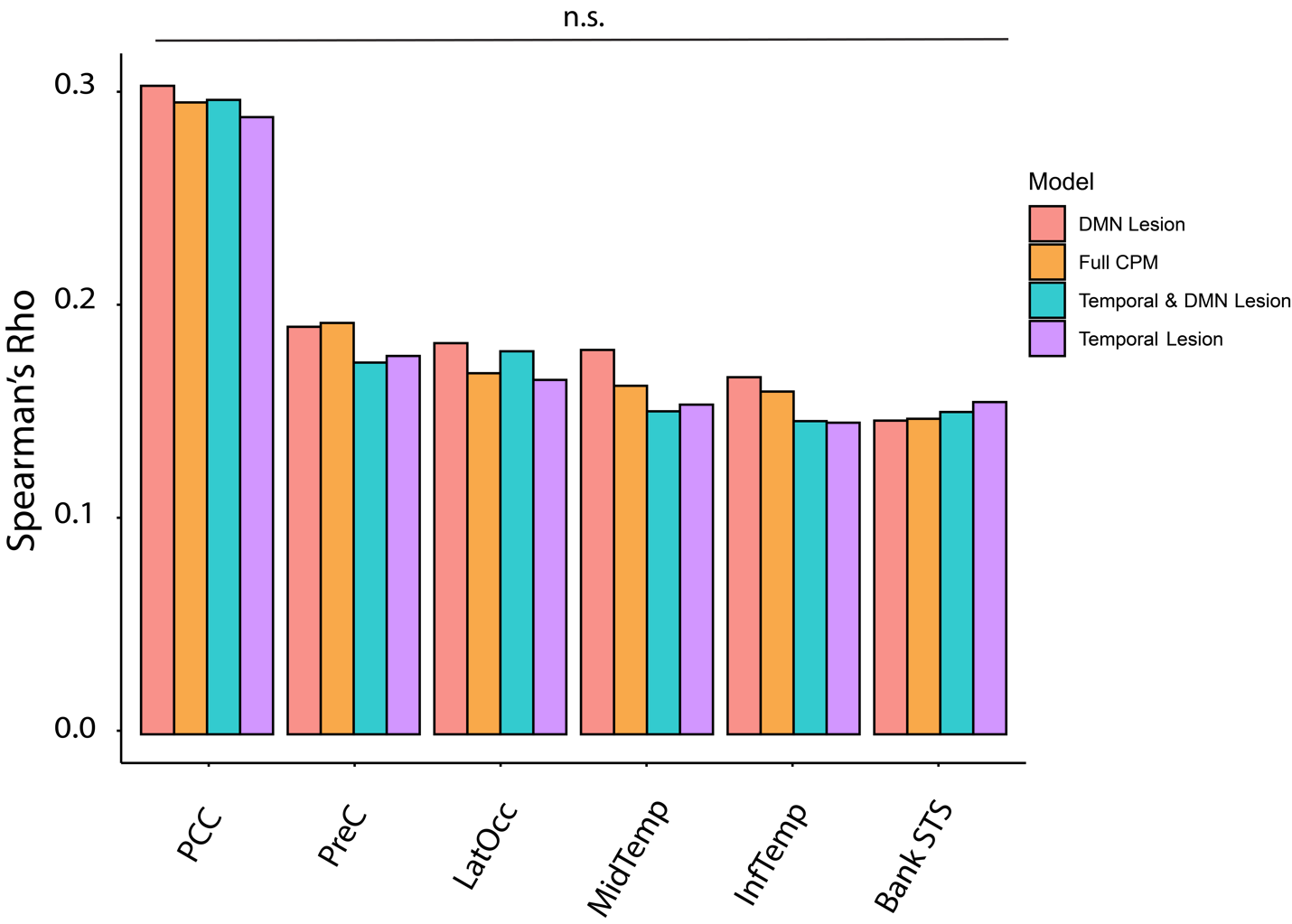
**

Supplemental Figure 5. Impact of lesioning of temporal lobe and default mode network (DMN)-associated nodes on tau prediction performance. Bar plots show model accuracy (spearman’s rho) following exclusion of respective nodes. n.s. = no significance (p>0.05) between pairwise models for each region using a Steiger’s z-test.

**
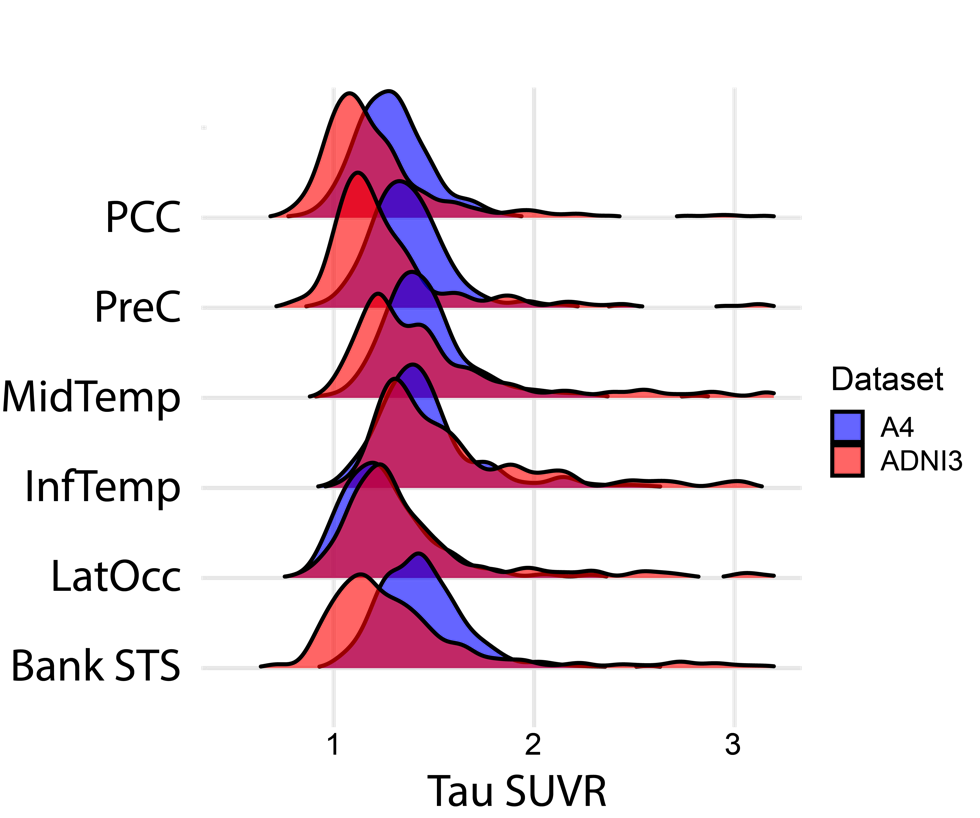
**

Supplemental Figure 6. Tau SUVR distributions in A4 and ADNI3 in amyloid-positive participants. Kernel density plots represent within-dataset probabilities of the SUVR distribution. PCC = posterior cingulate, PreC = precuneus, MidTemp = middle temporal, InfTemp = inferior temporal, LatOcc = lateral occipital, Bank STS = Bank of the Superior Temporal Sulcus

**
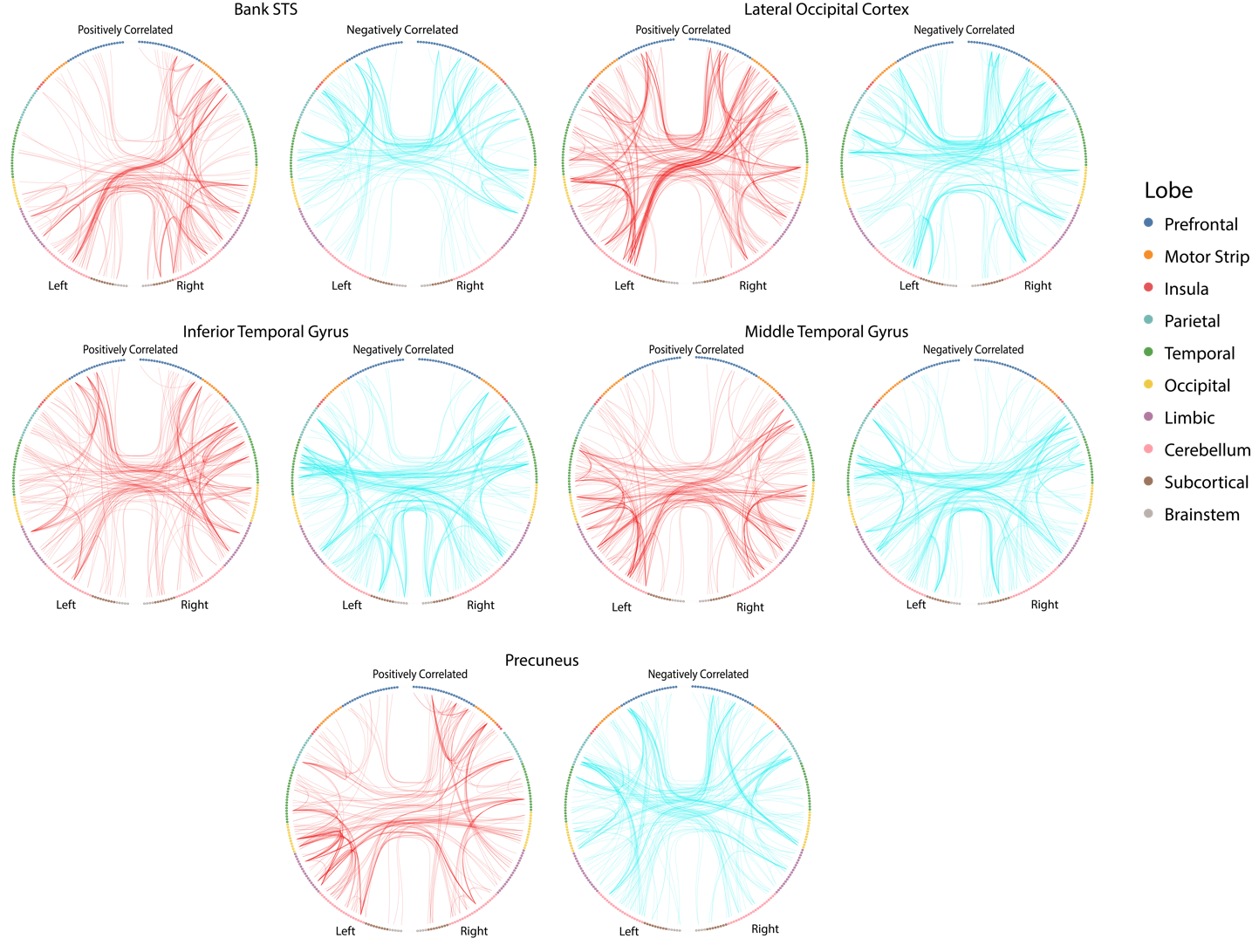
**

Supplemental Figure 7. Circle plots for A4 significant models. Each circle represents a node from the Shen-268 atlas, grouped according to their respective region. Edges are depicted by lines between each node pair. Plot with red lines shows edges that were positively correlated with PCC tau, while the plot with blue lines indicates edges that were negatively correlated with PCC tau. Only edges from the top 5% most predictive nodes (by degree) are displayed, based on edges significant in > 3/5 folds across > 600/1000 connectome-based predictive modeling (CPM) iterations.

**
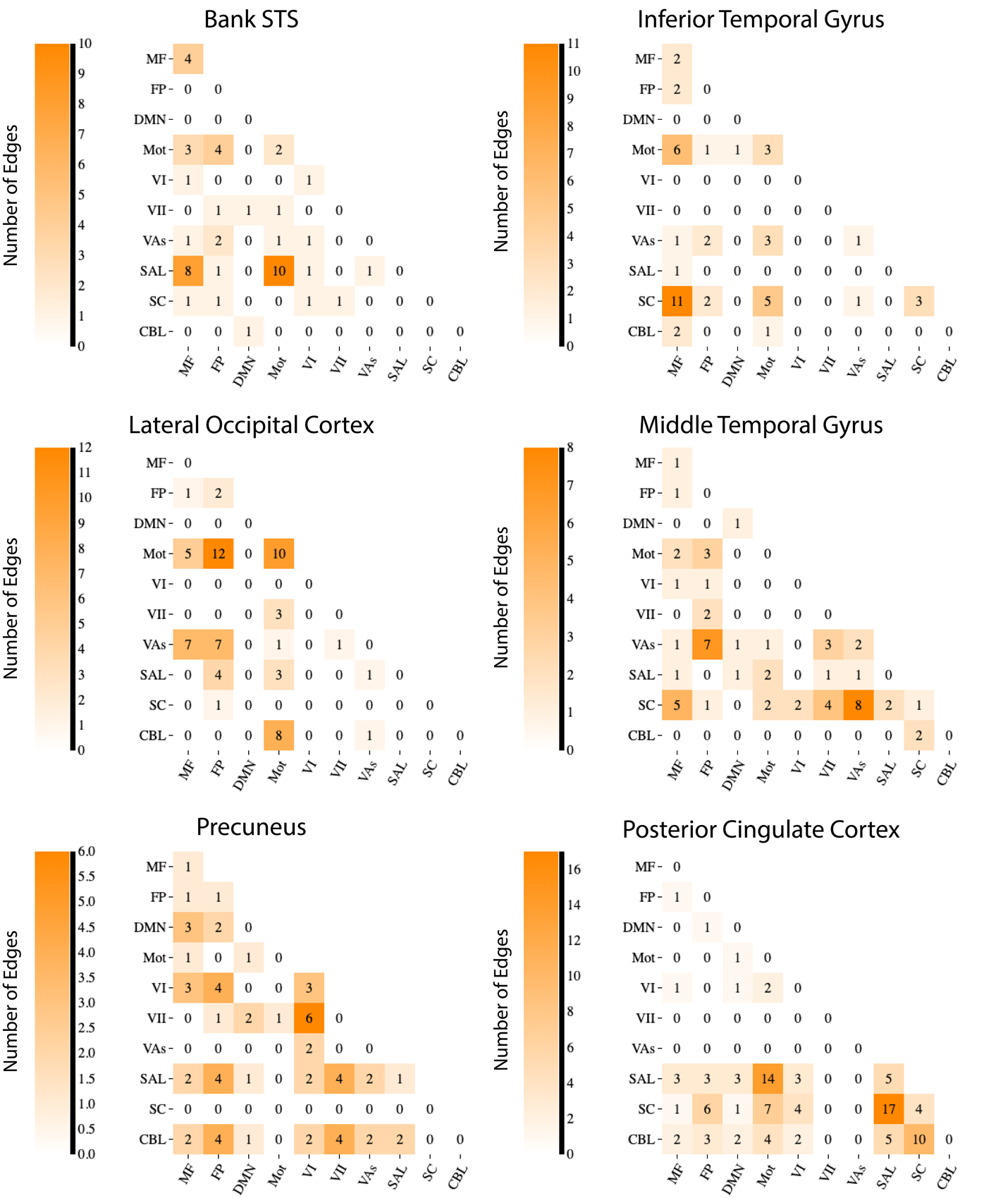
**

Supplemental Figure 8. Matrices showing distribution of predictive edges for each model, with nodes grouped by 10 canonical networks. Only edges from the top 5% most predictive nodes (by degree) are displayed, based on edges significant in > 3/5 folds across > 600/1000 connectome-based predictive modeling iterations. MF = mediofrontal; FP = frontoparietal; DMN = default mode network; Mot = Motor; VI = primary visual cortex; VII = secondary visual cortex; VAs = visual association; SAL = Salience; SC = subcortical; CBL = cerebellar.

| Node (shen atlas) | # pt missing | TAL X | TAL Y | TAL Z | MNIX | MNIY | MNIZ | BA region |
| --- | --- | --- | --- | --- | --- | --- | --- | --- |
| 2 | 200 | 9 | 14 | -14 | 10 | 18 | -19 | Right-OrbFrontal (11) |
| 4 | 247 | 15 | 29 | -20 | 16 | 34 | -23 | Right-OrbFrontal (11) |
| 51 | 130 | 26 | 6 | -31 | 27 | 12 | -40 | Right-Temporalpole (38) |
| 60 | 127 | 30 | -4 | -35 | 31 | 0 | -45 | Right-InfTempGyrus (20) |
| 107 | 58 | 45 | -49 | 0 | 46 | -49 | -6 | Right-Fusiform (37) |
| 131 | 157 | 5 | -24 | -32 | 6 | -22 | -42 | Outside defined Bas |
| 135 | 87 | -18 | 14 | -16 | -18 | 19 | -21 | Left-OrbFrontal (11) |
| 136 | 518 | -6 | 14 | -18 | -6 | 18 | -23 | Left-OrbFrontal (11) |
| 137 | 104 | -8 | 34 | -20 | -8 | 39 | -21 | Left-OrbFrontal (11) |
| 189 | 282 | -23 | 4 | -31 | -23 | 9 | -39 | Left-Temporalpole (38) |
| 196 | 150 | -49 | -20 | -22 | -52 | -18 | -29 | Left-InfTempGyrus (20) |
| 202 | 145 | -29 | -9 | -32 | -30 | -6 | -41 | Left-Parahipp (36) |
| 249 | 52 | -34 | -53 | -43 | -35 | -50 | -54 | Outside defined BAs |
| 252 | 120 | -44 | -50 | -34 | -46 | -47 | -44 | Outside defined BAs |
| 268 | 111 | -5 | -21 | -27 | -7 | -19 | -37 | Outside defined BAs |

Supplementary Table 1. List of nodes with greater than 50 participants (of the total 1490 participants) with signal dropout. Nodes listed above were removed from connectivity matrices prior to all analyses.

| ROI | BIC  1-component | BIC  2-component | Delta BIC | Abnormal Component mixing proportion, (estimated n) |
| --- | --- | --- | --- | --- |
| Inferior Parietal | -319 | -448 | -129 | 0.133 (45) |
| Fusiform | -455 | -550 | -95 | 0.172 (59) |
| Transverse Temporal | 144 | 54.6 | -89.4 | NA |
| Inferior Temporal | -418 | -501 | -83 | 0.184 (63) |
| Rostral Middle Frontal | -284 | -361 | -77 | 0.027 (9) |
| Superior Parietal | -308 | -382 | -74 | 0.079 (27) |
| Isthmus Cingulate | -282 | -356 | -74 | 0.055 (18) |
| Caudal Middle Fontal | -240 | -303 | -63 | 0.039 (13) |
| Precuneus | -372 | -434 | -62 | 0.081 (27) |
| Middle Temporal | -378 | -431 | -53 | 0.173 (59) |
| Bank STS | -361 | -405 | -44 | 0.075 (25) |
| Lateral Occipital | -346 | -387 | -41 | 0.122 (41) |
| Amygdala | -222 | -260 | -38 | 0.241 (82) |
| Supramarginal | -50.1 | -86.3 | -36.2 | 0.137 (47) |
| Superior Frontal | -488 | -519 | -31 | 0.105 (36) |
| Paracentral | -385 | -405 | -20 | 0.017 (6) |
| Frontal Pole | -165 | -182 | -17 | NA |
| Cuneus | 273 | 262 | -11 | 0.022 (7) |
| Parstriangularis | -476 | -486 | -10 | NA |
| Posterior Cingulate | -441 | -448 | -7 | 0.123 (42) |
| Parahippocampal | -380 | -387 | -7 | 0.065 (22) |
| Lingual | -148 | -154 | -6 | 0.046 (16) |
| Precentral | -515 | -520 | -5 | 0.022 (8) |
| Parsorbitalis | -542 | -546 | -4 | 0.037 (13) |
| Entorhinal | -291 | -295 | -4 | 0.111 (40) |
| Parsopercularis | -17.5 | -18.5 | -1 | 0.02 (7) |
| Temporal Pole | -434 | -434 | 0 | NA |
| Rostral Anterior Cingulate | -273 | -273 | 0 | NA |
| Postcentral | -294 | -293 | 1 | NA |
| Caudal Anterior Cingulate | -441 | -437 | 4 | NA |
| Superior Temporal | -258 | -251 | 7 | NA |
| Medial Orbitofrontal | -590 | -583 | 7 | NA |
| Lateral Orbitofrontal | -404 | -395 | 9 | NA |
| Pericalcarine | -454 | -444 | 10 | NA |
| Insula | -351 | -336 | 15 | NA |

Supplementary Table 2. For each region of interest (ROI) from A4 (n=342), the table reports the BIC of the one-component and two-component Gaussian mixture models, along with the delta BIC (2-component – 1 component) and mixing proportion of the abnormal/elevated tau component. We do not report mixing proportions for models where 1-component>2-component, and for regions with abnormal 2-component GMM: pars triangularis (due to approximately equal Gaussian means between components), the transverse temporal cortex (where the abnormal component AUC exceeded the normal component), and frontal pole (abnormally high mixing proportion of the abnormal component).

| ROI | Spearman’s Rho (edge p = 0.05) | Spearman’s Rho  (edge p = 0.01) |
| --- | --- | --- |
| Posterior Cingulate | 0.295 | 0.282 |
| Precuneus | 0.192 | 0.186 |
| Lateral Occipital | 0.169 | 0.155 |
| Middle Temporal | 0.163 | 0.153 |
| Inferior Temporal | 0.160 | 0.142 |
| Bank STS | 0.147 | 0.134 |
| Temporal -ROI | 0.142 | 0.121 |
| Superior Frontal | 0.130 | 0.142 |
| Inferior Parietal | 0.095 | 0.103 |
| Supramarginal | 0.082 | 0.087 |
| Amygdala | 0.060 | 0.040 |
| Fusiform | 0.060 | 0.059 |
| Superior Parietal | 0.050 | 0.050 |
| Parahippocampal | 0.046 | 0.045 |
| Entorhinal | 0.027 | 0.028 |

Supplementary Table 3. Comparison of model performance metrics between models with feature selection threshold of p = 0.05 and p = 0.01 for n = 342 subjects (amyloid elevated only).

| ROI | Bilateral ROI | Right-Hemisphere ROIs | Left-Hemisphere ROIs |
| --- | --- | --- | --- |
| Posterior Cingulate | 0.293 | 0.294 | 0.280 |
| Precuneus | 0.193 | 0.184 | 0.216 |
| Lateral Occipital | 0.170 | 0.117 | 0.140 |
| Middle Temporal | 0.167 | 0.148 | 0.097 |
| Inferior Temporal | 0.161 | 0.147 | 0.118 |
| Bank STS | 0.150 | 0.146 | 0.103 |

Supplementary Table 4. Model performance (median spearman’s) using z-scored tau PET SUVRs as model inputs for bilateral, right and left-specific hemispheric ROIs. Z-scores were generated by first developing a linear regression of model of age and sex against tau SUVR in the healthy control (LEARN) population. This normative model was then applied to the amyloid-positive individuals (n=342), and their deviations from the predicted values were used to generate z-scores.
